# Child Weight Gain Trajectories Linked To Oral Microbiota Composition

**DOI:** 10.1038/s41598-018-31866-9

**Published:** 2018-09-19

**Authors:** Sarah J. C. Craig, Daniel Blankenberg, Alice Carla Luisa Parodi, Ian M. Paul, Leann L. Birch, Jennifer S. Savage, Michele E. Marini, Jennifer L. Stokes, Anton Nekrutenko, Matthew Reimherr, Francesca Chiaromonte, Kateryna D. Makova

**Affiliations:** 1 Center for Medical Genomics, Penn State University, University Park, PA 16802 USA; 2 Department of Biology, Penn State University, University Park, PA 16802 USA; 3 Department of Biochemistry and Molecular Biology, Penn State University, University Park, PA 16802 USA; 4 Department of Mathematics, Politecnico di Milano, Piazza Leonardo da Vinci, 32, Milano, 20133 Italy; 5 Department of Pediatrics, Penn State College of Medicine, 500 University Drive, Hershey, PA 17033 USA; 6 Department of Foods and Nutrition, 176 Dawson Hall, University of Georgia, Athens, GA 30602 USA; 7 Center for Childhood Obesity Research, Penn State University, University Park, PA 16802 USA; 8 Department of Nutritional Sciences, Penn State University, University Park, PA 16802 USA; 9 Department of Statistics, Penn State University, University Park, PA 16802 USA; 10 EMbeDS, Sant’Anna School of Advanced Studies, Piazza Martiri della Libertà, 33, Pisa, 56127 Italy; 11 Present Address: Genomic Medicine Institute, Lerner Research Institute, Cleveland Clinic, Cleveland, OH 44195 USA

## Abstract

Gut and oral microbiota perturbations have been observed in obese adults and adolescents; less is known about their influence on weight gain in young children. Here we analyzed the gut and oral microbiota of 226 two-year-olds with 16S rRNA gene sequencing. Weight and length were measured at seven time points and used to identify children with rapid infant weight gain (a strong risk factor for childhood obesity), and to derive growth curves with innovative Functional Data Analysis (FDA) techniques. We showed that growth curves were associated negatively with diversity, and positively with the Firmicutes-to-Bacteroidetes ratio, of the oral microbiota. We also demonstrated an association between the gut microbiota and child growth, even after controlling for the effect of diet on the microbiota. Lastly, we identified several bacterial genera that were associated with child growth patterns. These results suggest that by the age of two, the oral microbiota of children with rapid infant weight gain may have already begun to establish patterns often seen in obese adults. They also suggest that the gut microbiota at age two, while strongly influenced by diet, does not harbor obesity signatures many researchers identified in later life stages.

## Introduction

One in three children in the United States are overweight or obese^1^. An association between this phenotype and the microbiota (a collection of microorganisms)^2^ of the host has been shown in multiple studies (reviewed in^3^). The most striking evidence of this phenomenon comes from mice: germ-free mice inoculated with the microbiota of obese mice were shown to develop obesity, whereas their littermates inoculated with the microbiota of lean mice did not^4,5^. Relative to lean mice, obese mice displayed an increase in Firmicutes and a decline in Bacteroidetes^6^. Similar patterns have been observed in humans; the gut microbiota of obese adult and adolescent individuals also displayed low diversity^7,8^ and an elevated Firmicutes-to-Bacteroidetes (F:B) ratio^7–9^. However, these gut microbiota alterations are not universally linked to obesity^3,10,11^. Study results frequently depend on the methods used (e.g. 16S variable region analyzed, sequencing platform, or computational pipeline) and a number of co-factors that can affect gut microbiota − diet^12,13^, exposure to antibiotics^14^, the use of non-steroid anti-inflammatory drugs^15^, host genetics^16–18^, and age^19^. Some published reports investigated the connection between the gut microbiota and binary differences in outcomes of growth in young children^8^. Importantly though, to our knowledge, no prior study explored the connection between the gut microbiota and the actual temporal trajectories of weight gain in young children. In particular the role of the gut microbiota during the earliest stages of weight gain − for neonates (from birth to one month), infants (1–12 months), and toddlers (12–24 months) − remain underexplored. Weight gain trajectories vary greatly among children^20^, and thus may represent a more informative phenotype than a binary outcome (stunted vs. normal, or obese vs. normal) and may increase statistical power in detecting associations between weight gain and microbiota.

In contrast to the many studies concerning the gut microbiota, very few studies explored the *oral* microbiota and its relationship with weight gain. These latter studies stem from the observed relationship between periodontal disease prevalence and obesity in adults^21,22^. There is a body of literature investigating differences in the oral microbiota in relation to periodontal disease and dental caries^23–27^. Several studies directly investigating the relationship between oral microbiota and obesity, again through the lens of oral health, found differences in the oral microbiota composition of obese vs. lean adults and adolescents^28,29^. One such study of oral microbiota in adults pointed to the Bacteroidetes species, *Tannerella forsythia*, as having different prevalence in healthy-weight, overweight, and obese groups^30^. In adult women’s oral microbiota it was found that *Selenomonas noxia*, a Firmicutes species, can predict overweight status^29^. In adolescents’ oral microbiota, Zeigler and colleagues documented an increase of both Firmicutes and Bacteroidetes in obese compared to normal-weight individuals, with no significant difference in the abundance between these two phyla^28^. As with the gut microbiota, to our knowledge, there has been no study investigating the relationship of the oral microbiota with the temporal growth trajectories of young children.

Early childhood is a time marked by dramatic microbiota changes^31–35^. A pivotal seeding of the child’s microbiota occurs during delivery. Infants delivered vaginally have oral and gut microbiota similar to their mothers’ vaginal microbiota^35–37^, while infants delivered via Cesarean section have oral and gut microbiota that resemble their mothers’ skin microbiota^38^. Factors that could influence the composition of this influx are the mother’s weight gain^39^, diabetes^40^, and smoking during pregnancy^41^. Nonetheless, differences in gut microbiota due to delivery mode might be erased by the mounting effects of other factors as early as six weeks after birth^35^. Diet is one such factor. The gut microbiota differs between breast- and formula-fed infants^35^, and the first year after birth also comprises other diet transitions affecting gut and oral microbiota^33,42^. For instance, high-fat and high-carbohydrate diets have been associated with high and low infant gut microbiota F:B ratios, respectively^43^. Aside from diet, antibiotics have been shown to influence weight. For instance, exposure to antibiotics in the first two years was associated with higher weight in later childhood^14,44–46^, but this link has been recently questioned^47,48^. Additionally, the use of acid-reducing drugs (proton pump inhibitors and histamine antagonists) have been associated with decreased gut microbiota diversity in adults and premature infants (<34 weeks gestation)^49–51^. These drugs are widely prescribed, however we are only now beginning to understand their potential effects on the adult microbiota, and know even less about their influence on a child’s developing microbiota. Early life transitions and their effects on the gut microbiota are often described as “chaotic”^32^ perhaps because they happen over a short period of time, or because there is not a specific order to their succession^33^. Nonetheless, within the first several years of life the children’s gut microbiota converge on a more “adult-like” composition^18,19,34,52^.

In this report we present results from comprehensive analyses of microbiota composition with a wealth of clinical, anthropometric, demographic, and behavioral variables collected on 236 mother-child dyads enrolled in the Intervention Nurses Start Infants Growing on Healthy Trajectories (INSIGHT) study^53^. Our goals for this study were to: (a) model children growth curves and examine their associations with oral and gut microbiota; (b) assess whether oral and gut microbiotas differ between children with rapid vs. non-rapid infant weight gain; (c) investigate whether there is any relationship between a mother’s oral microbiota and her child’s weight trajectory, and (d) analyze whether diet and other factors have an impact on oral and gut microbiota and/or the growth of the child. Our results demonstrated significant associations between the oral microbiota, as established at age two, and rapid weight gain during the first two years after birth. We also provided evidence for significant effects of diet on the gut microbiota, which may modulate growth trajectories in children. Finally, we pioneered the use of functional data analysis (FDA) techniques in microbiota studies; these techniques allow us to fully leverage the complex, multifaceted phenotypic dynamics of the surveyed individuals.

## Results

### Oral and gut microbiota profiling

From the 279 families recruited in INSIGHT that had complete longitudinal anthropometric and behavioral measurements over the first two years after birth^53^, we collected samples from 236 mother-child dyads (specifically, oral samples for 229 mothers and 225 children, and stool samples for 200 children; see Supplementary Fig. S1 for a graphical summary and a flowchart) at the child’s two-year clinical research lab visit. Key characteristics of the study population are summarized in Table 1. For our oral and stool samples, we sequenced the variable regions 3 and 4 of the 16S rRNA gene and analyzed the data with custom Galaxy^54^ workflows (see Methods). After filtering and controlling for quality (see Methods), we retained 215 maternal oral, 214 child oral, and 189 child stool samples (Supplementary Fig. S1). 151,771,821 total reads were retained after quality control with an average of 243,224 reads per sample and a range of 100,626–1,898,073 reads among samples. Each read was classified using the GreenGenes database, and, for each sample, ecological diversity measurements (α-diversity − a measure of species richness and evenness within a single sample, and β-diversity − a measure of species differences between samples^55^, see Methods) were calculated from rarefied phylum level classifications using the Vegan package^56^.

**Table 1:** Description of the study population.

	Children with rapid infant weight gain (CWG^a^ ≥ 0), N = 104	Children without rapid infant weight gain (CWG < 0), N = 122)
**Children**		
Gender: Number (%) of males	53 (51)	67 (55)
Gestational age: weeks (S.D.)	39.59 (1.17)	39.46 (1.22)
Birth weight: kg (S.D.)	3.44 (0.44)	3.41 (0.41)
Birth length: cm (S.D.)	51.03 (2.39)	50.73 (2.26)
Mothers		
Age (years): mean (S.D.)	28.87 (4.40)	29.20 (4.75)
Pre-pregnancy BMI: mean (S.D.)	25.78 (5.55)	25.41 (5.03)
Gestational Weight Gain: mean (S.D)	15.73 (5.91)	14.46 (6.33)
**Annual household income N (%)**		
<$10,000	3 of 99 (1.4)	4 of 118 (1.8)
$10,000–$24,999	5 of 99 (2.3)	9 of 118 (4.2)
$25,000–$49,999	12 of 99 (5.5)	10 of 118 (4.6)
$50,000–$74,999	29 of 99 (13.4)	34 of 118 (15.7)
$75,000–$99,999	26 of 99 (12)	22 of 118 (10.1)
$100,000 or more	24 of 99 (11)	39 of 118 (18.0)
**Ethnicity** ^**b**^ **N (%)**		
Black	4 (3.9%)	5 (4.1%)
White	98 (94%)	111 (91%)
Native Hawaiian or Pacific Islander	0%	1 (0.8%)
Asian	1 (1%)	4 (3.3%)
Other	1 (1%)	1 (0.9%)
**Maternal Education N (%)**		
HS graduate or less	10 (9.6%)	6 (4.9%)
Some college	28 (26.9%)	27 (22.1%)
College graduate	40 (38.5%)	51 (41.8%)
Graduate degree +	26 (25.0%)	38 (31.2%)

^a^CWG is conditional weight gain (see text for more details).

^b^Ethnicity was self-reported.

While variability among individuals was high, oral and gut microbiota samples showed distinct differences in composition at the phylum level: namely, abundance of Fusobacteria and absence of Verrucomicrobia in the oral microbiota, and an opposite pattern in the gut microbiota (Supplementary Fig. S2A). These results are largely consistent with prior studies exploring the composition of healthy, adult gut^57^ and oral^58^ microbiota. In general, the oral microbiota (Supplementary Fig. S2A) was comprised largely of Firmicutes, with moderate levels of Proteobacteria, Actinobacteria, Bacteroidetes, and Fusobacteria. The gut microbiota presented high levels of both Firmicutes and Bacteroidetes, with lower levels of Verrucomicrobia, Actinobacteria, and Proteobacteria (Supplementary Fig. S2A). Both types of microbiota had low levels of bacteria belonging to the TM7 and ‘Other’ phyla. Using a test of equal proportions (Pearson’s chi-squared test), significant differences were found in the proportion of Bacteroidetes (p = 1.1 × 10^−14^), Firmicutes (p = 2.9 × 10^−3^), Proteobacteria (p = 8.7 × 10^−6^), and Verrucomicrobia (p = 2.0 × 10^−5^) between the child gut microbiota and the mother oral microbiota. Additionally, there were significantly different proportions of Bacteroidetes (p = 2.2 × 10^−6^), Proteobacteria (p = 7.1 × 10^−6^), and Verrucomicrobia (p = 2.2 × 10^−5^) between child gut microbiota and child oral microbiota. However, no significant differences in the proportions of any bacterial phyla were observed between maternal and child oral samples. Using non-metric multidimensional scaling (NMDS) to visualize the distances among the three microbiota types (Supplementary Fig. S2B), we found that there was some overlap between oral and gut microbiota communities, but gut samples largely clustered together and away from oral samples (mother and child), and children’s oral samples formed a tighter cluster than maternal oral samples. These results corroborate the significant differences in proportions between child gut/mother oral and child gut/child oral microbiotas.

### Growth curves and their relationship with the microbiota

**Figure 1:**
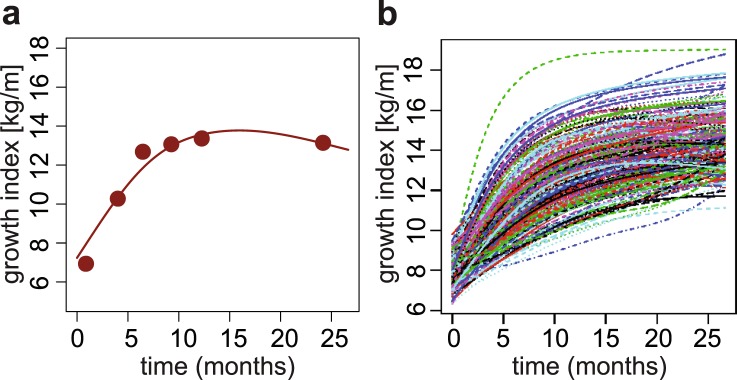
Growth curves construction. (**a**) Example growth curve. Points: observed weight-for-length ratios (i.e. growth indexes); line: estimated growth curve. (**b**) Final aligned growth curves for all children studied.

Since the outcome measurement recommended by American Academy of Pediatricians for children under two years of age is weight-for-length^59^, growth curves were constructed based on this ratio (later called *growth index*) measured at seven time points during the first two years after birth (see Methods). This set of longitudinal observations (Fig. 1a and Supplementary Fig. S3) was then processed with FDA techniques^60^: the growth indexes of all children were pooled to estimate population-level curve parameters (mean and covariance functions), and smooth individual growth curves were constructed forming best linear unbiased predictors of the missing segments (see Methods). These curves were monotonously increasing (Supplementary Fig. S3B), highlighting fast growth rate over the first two years after birth. Because each child may hit growth spurts at a slightly different age^20^, we aligned the curves based on their dominant shapes (Fig. 1b). The alignment procedure had only minor effects on the curves (compare Supplementary Fig. S3B and Fig. 1b) but allowed us to focus on variation in the amplitude of the curves (i.e. growth index on the vertical axis), while reducing variation in their temporal phasing (i.e. time on the horizontal axis). The functional regressions we utilized in this study can be thought of as a more general, non-parametric alternative to a mixed effect model − which captures the longitudinal nature of the data, and in fact does so without making specific assumptions on the form of the mean relationship, and of the intraindividual dependence structure of the observations. This approach is more general and can be more effective than classic multivariate mixed model analyses when the data exhibit complex temporal dynamics^61^.

**Figure 2:**
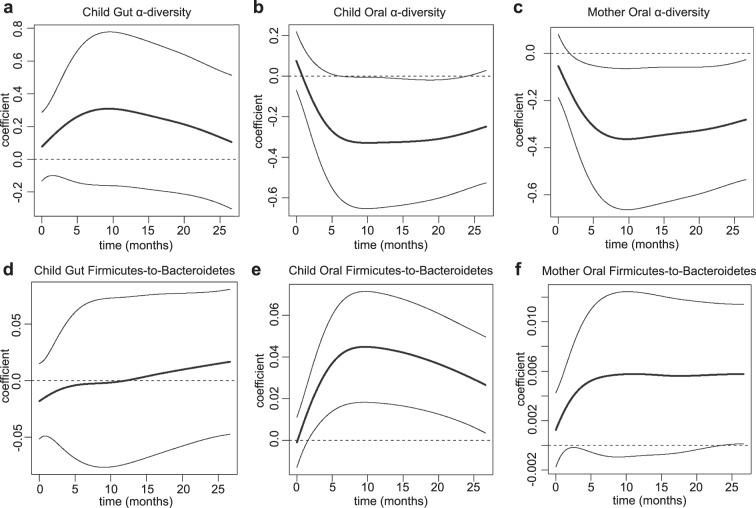
Oral and Gut microbiota’s relationships with growth curves. Estimated regression coefficient curves expressing the associations of growth curves with (**a**) children’s gut α-diversity, (**b**) children’s oral α-diversity, (**c**) mothers’ oral α-diversity, (**d**) children’s gut Firmicutes-to-Bacteroidetes ratio, (**e**) children’s oral Firmicutes-to-Bacteroidetes ratio and (**f**) mothers’ oral Firmicutes-to-Bacteroidetes ratio. Each curve is accompanied by a point-wise confidence band^92^.

To evaluate associations between children’s weight gain and the microbiota information, we started by applying FDA regression techniques (see Methods). Using children’s growth curves (Fig. 1b) as the response we ran functional regressions, each with a single scalar predictor − children’s oral α-diversity, oral F:B ratio, gut α-diversity or gut F:B ratio; and mothers’ oral α-diversity or oral F:B ratio. These functional regressions produce regression *coefficient curves* − as opposed to scalar regression coefficients. Estimated coefficient curves above/below the zero line suggest negative and positive associations respectively. Significance of associations can be measured through p-values (see Supplementary Table S1) and also gleaned by whether the zero line is contained within a curve’s 95% confidence band. Our results suggest that low microbial diversity and high content of Firmicutes relative to Bacteroidetes in the mouth of a two-year-old are markers of elevated growth indexes during the first two years after birth − the estimated coefficient curves were negative and positive, respectively, with confidence bands that did not contain the zero line (Fig. 2b,e), and p = 4.1 × 10^−2^ for diversity and p = 1.5 × 10^−3^ for F:B ratio (Choi test^62^, Supplementary Table S1). Conversely, estimated coefficient curves for the gut were closer to the zero line (Fig. 2a,d) and p-values were large (p = 0.27 for diversity and p = 0.72 for F:B ratio, Choi test^62^, Supplementary Table S1), suggesting that α-diversity and F:B ratio in the gut of a two-year-old are not significantly associated with his/her growth trajectory from birth to two years. Interestingly, α-diversity, but not the F:B ratio, of mothers’ oral microbiota were significantly associated with their children’s growth curves (p = 1.8 × 10^−2^, Choi test^62^, Fig. 2c,f, and Supplementary Table S1). In fact, the α-diversities of children’s and mothers’ oral microbiota were significantly correlated (R = 0.20, p = 3.0 × 10^−3^), and the corresponding estimated regression coefficient curves had similar shapes (Fig. 2b,c).

### Rapid infant weight gain and its relationship with the microbiota

To complement the analyses based on growth curves, we associated the binary quantification of rapid vs. non-rapid infant weight gain, as defined by Conditional Weight Gain (CWG) scores, a measure commonly used in pediatric research^63,64^, to the microbiota information. Children’s CWG scores were computed from weight gain between birth and six months, corrected by length at these two time points^63,64^ (see Methods). A CWG score ≥0 indicates weight gain that was faster than average and was used to define rapid infant weight gain^64^ − a predictor of obesity later in life^65^. In our cohort, 104 children had rapid infant weight gain between birth and six months (CWG ≥ 0) and 122 did not (CWG < 0). Notably, the former had a significantly greater weight at two years of age than the latter (p = 4.6 × 10^−13^, Mann-Whitney one-tailed t-test; Supplementary Fig. S4).

**Figure 3:**
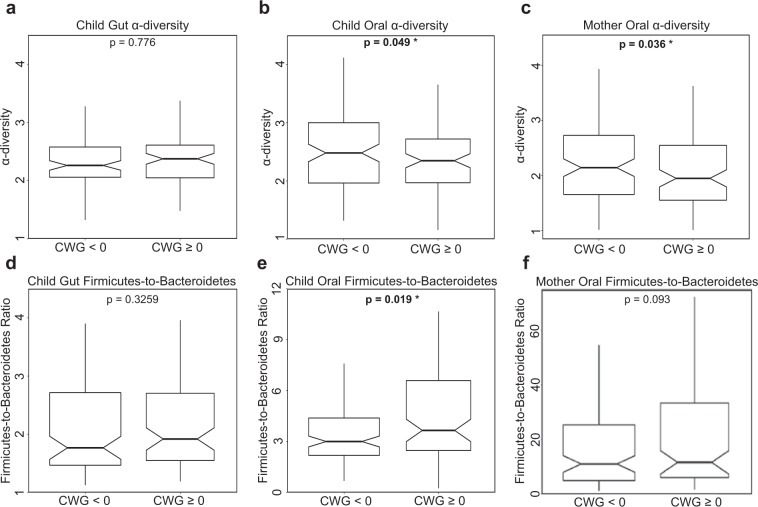
Oral and Gut microbiota’s relationships with conditional weight gain. Notched box-plots contrasting α-diversity and Firmicutes-to-Bacteroidetes (F:B) ratio in two-year-old children with rapid (CWG ≥ 0) vs. non-rapid (CWG < 0) weight gain, and in their mothers. (**a,d**) for the *gut* microbiota in children with rapid (N = 90) vs. non-rapid (N = 99) weight gain; (**b,e**) for the *oral* microbiota in children with rapid (N = 97) vs. non-rapid infant weight gain (N = 117); (**c,f**) for the oral microbiota in mothers of children with rapid infant weight gain (N = 102) vs. mothers of children without rapid infant weight gain (N = 113). All p-values were obtained using one-tailed Mann-Whitney U tests, significant p-values are shown in bold. Outliers were not plotted but were included in the statistical tests.

To test for associations between CWG scores and microbiota, we assessed whether the microbiota of children with rapid infant weight gain possessed the obesity signatures previously found in the gut microbiota of older obese children and adults (see Introduction) – namely, lower diversity and higher F:B ratio than non-rapid infant weight gain children. This was indeed the case for the oral microbiota (p = 0.049 and p = 0.019, respectively, one-tailed Mann-Whitney U test; Fig. 3b,e) but, interestingly, not for the gut microbiota (p = 0.78 and p = 0.33, respectively; Fig. 3a,d). Next, we tested whether mothers of children with rapid vs. non-rapid infant weight gain had significantly different oral microbiota α-diversities and F:B ratios. Again we found that diversity was significantly lower in mothers of children with rapid infant weight gain (p = 0.036, Fig. 3c), but the F:B ratio was not significantly higher (p = 0.093, Fig. 3f). The patterns in these analyses were consistent with those in the growth curves analyses, but the latter provided stronger significance assessments (Supplementary Table S1), demonstrating the effectiveness of FDA techniques − which incorporate longitudinal information in a richer and more nuanced fashion.

### Potential co-factors and their roles

Many factors could affect children’s microbiota and their relationships with weight gain. Based on the anthropometric and behavioral data at our disposal^53^, we considered a total of 17 potential confounders − gender, exposure to antibiotics and acid-reducing medication during the first two years after birth, delivery mode (vaginal vs. C-section), INSIGHT intervention^53^, maternal gestational diabetes, gestational weight gain, smoking during pregnancy, family income, and eight diet categories at age two (see Methods). The effect of each non-diet factor on microbiome measurements (children’s oral and gut α-diversity and F:B ratio at two years of age) were first tested individually and were found to be non-significant (Supplementary Table S2). Two multiple regressions on diet-related variables confirmed strong effects of diet on both gut α-diversity and gut F:B ratio (overall p = 2.6 × 10^−3^ for α-diversity regression and p = 1.7 × 10^−7^ for the F:B ratio regression; Supplementary Table S3). Two multiple regressions on diet-related variables on oral F:B ratio and α-diversity were not significant (Supplementary Table S3). Thus, diet at age two had marked effects on the gut microbiota (on both α-diversity and F:B ratio) at the same age but not on oral microbiota.

We also performed variable selection for multiple regressions comprising all 17 potential covariates mentioned in the previous paragraph (Supplementary Tables S2 and S3). Notably, only diet-related variables were selected by the procedure, and only in regressions for the gut microbiota (no covariates were retained in regressions for the oral microbiota). Fitting regressions restricted to the selected diet-related variables, we explained as much as 21% of the variability in gut F:B ratio (overall p = 9.5 × 10^−9^), with vegetable and meat consumption as significant positive and negative predictors, respectively, and a more modest 5.9% of the variability in gut α-diversity (overall p = 3.8 × 10^−3^), with vegetable and fruit consumption as significant negative and positive predictors, respectively (Table 2).

**Table 2:** Associations detected in multiple linear regressions for children’s gut microbiotas’ Firmicutes-to-Bacteroidetes ratio and α-diversity.

Response	Covariate	Coefficient estimate	T-value*	P-value	Adjusted R-squared
Gut F:B ratio	vegetables	15.0	6.21	5.08 × 10^−9^	
	meats	−18.8	−3.28	1.28 × 10^−3^	
*Overall model*				9.45 × 10^−9^	20.79%
Gut α-diversity	vegetables	−0.0769	−3.22	1.57 × 10^−3^	
	fruit	0.0543	2.63	9.52 × 10^−3^	
*Overall model*				3.76 × 10^−3^	5.94%

Each multiple regression comprised 17 potential covariates, but only diet-related ones were retained by variable selection procedures. Coefficient estimates and significance (p-values) are shown, together with overall model significance and R-squared, for the regressions restricted to the selected diet-related covariates.

^*^Test statistic of a null hypothesis that a covariate’s coefficient is equal to zero.

Functional regressions of our growth curves on diet-related variables did not indicate any significant effects (p-values all above 0.090; Supplementary Table S4). Nevertheless, because of its strong effects on the gut microbiota at age two, diet may be a modulator of the gut microbiota’s relationship with weight gain. To assess this, we repeated the two functional regressions of growth curves on gut microbiota − one regression for α-diversity and one for F:B ratio − adding diet-related covariates with significant effects on the microbiota (from the previous variable selection procedure, see Table 2). Notably, gut α-diversity became a significant positive predictor of growth curves when considered together with diet (p = 0.020, Choi test^62^; Supplementary Table S5 and Supplementary Fig. S5A), while gut F:B ratio remained non-significant (p = 0.48, Choi test^62^; Supplementary Table S6 and Supplementary Fig. S5B).

### Weight gain and microbiota composition: Influential taxa

Next, we went beyond α-diversity and F:B ratio summary measures, which were computed at the coarse phylum level, and considered microbiota composition at a finer resolution to identify bacterial genera associated with a child’s weight gain. We computed *genus-level*, normalized abundances for the same 214 children’s oral microbiota, 189 children’s gut microbiota, and 215 mothers’ oral microbiota used for the analyses in the previous sections. Because many of these abundances were very low or highly collinear, we implemented a procedure to aggregate them into *taxonomic groups* (below we refer to these also as *bacterial groups*, or simply *groups*), leveraging the phylogeny of bacterial genera (see Methods; about 25% of the groups comprised just one bacterial genus, but 75% aggregated two or more genera that were either very scarce or highly correlated). We obtained 75, 77, and 79 taxonomic groups for children’s oral, children’s gut, and mothers’ oral microbiota, respectively (Supplementary Table S7). Based on the resulting aggregated abundances, and separately for the three microbiota, we used FLAME (Functional Linear Adaptive Mixed Estimation; a novel FDA methodology recently developed by our group)^62,66^ to identify taxonomic groups that were the best predictors of children’s growth curves (Supplementary Table S8).

**Figure 4:**
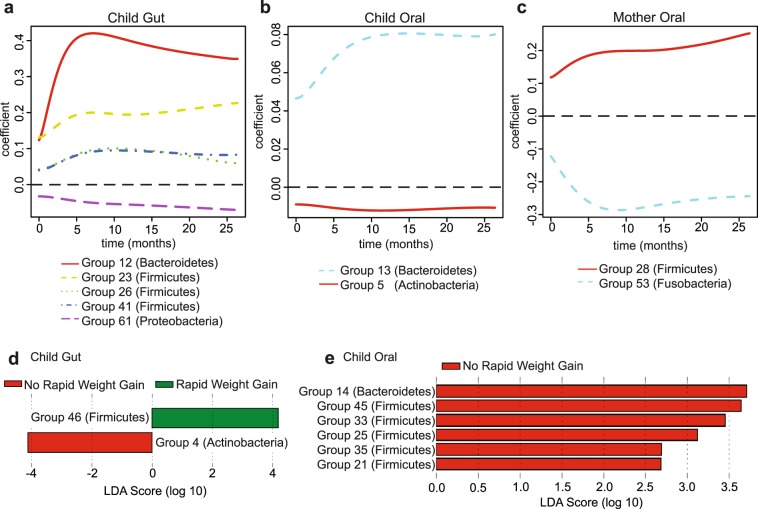
Identification of influential taxonomic groups. Estimated regression coefficient curves for taxonomic groups affecting growth curves, as identified by FLAME^66^ in **(a)** children’s gut microbiota, **(b)** children’s oral microbiota, **(c)** mothers’ oral microbiota. The zero line (dashed) corresponds to no effect. Linear Discriminant Analysis scores for taxonomic groups distinguishing children with rapid vs. non-rapid infant weight gain, as identified by LEfSe^67^ in **(d)** children’s gut microbiota, **(e)** children’s oral microbiota. See Supplementary Table S8 for a list of genera belonging to each group identified by FLAME and LEfSe.

In the children’s gut microbiota FLAME detected a group from the Proteobacteria phylum with a negative effect (Group 61), three Firmicutes groups with positive effects (Groups 23, 26, and 41) and one Bacteroidetes group with a positive effect (Group 12; Fig. 4a). In the children’s oral microbiota FLAME detected a group of Bacteroidetes (Group 13) and a group of Actinobacteria (Group 5) having, respectively, positive and negative effects (Fig. 4b). Two groups were detected in the mothers’ oral microbiota (Fig. 4c) − one Firmicutes group with a positive effect (Group 28) and one Fusobacteria group with a negative effect (Group 53).

We also used the Linear Discriminant Analysis (LDA) effect size tool, LEfSe^67^, to identify taxonomic groups that were most informative for separating children with rapid vs. non-rapid infant weight gain (Supplementary Table S8). In the children’s gut microbiota, LEfSe detected two discriminant bacterial groups; an Actinobacteria group associated with non-rapid infant weight gain and a Firmicutes group associated with rapid infant weight gain (Groups 4 and 46; Fig. 4d). In the children’s oral microbiota, LEfSe detected several discriminant groups associated with non-rapid infant weight gain, belonging to the Bacteroidetes and Firmicutes phyla (Groups 14, 21, 25, 33, 35, and 45; Fig. 4e). LEfSe did not identify any groups separating children with rapid vs. non-rapid infant weight gain in their mothers’ oral microbiota.

## Discussion

Our results demonstrated that, for our study population, a child’s *oral* microbiota, as analyzed at age two, was associated with weight gain during the first two years after birth. In particular, it displayed the decreased diversity and increased F:B ratio typically observed in the *gut* microbiota of obese adolescents and adults^7^. This conclusion was supported by sophisticated FDA techniques leveraging longitudinal information on weight gain in the first two years after birth, and confirmed by standard statistical tests based on a binary phenotype (rapid vs. non-rapid infant weight gain). Notably, links between the oral microbiota and a child’s weight gain trajectory have not been previously demonstrated for this age group, nor for a healthy population. These results suggest that the association between the oral microbiota and the temporal pattern of weight gain in early childhood might be stronger and more consequential than previously thought, and thus requires further characterization.

Why does the oral microbiota carry markers of rapid infant weight gain in children? Some studies have pointed to potential mechanisms linking periodontal disease and obesity, including increased oxidative stress^68^, low-grade systemic inflammation and insulin resistance^69,70^, and higher gingival crevicular fluid TNF-ɑ^22,71^. Goodson and colleagues^29^ hypothesized that the oral microbiota could (a) affect the gastrointestinal tract to increase metabolic efficiency, resulting in increased fat storage; (b) affect leptin or ghrelin levels, resulting in increased appetite and food consumption; and/or (c) affect TNF-α and adiponectin pathways, resulting in insulin resistance and increased fat storage. Gingival inflammation and decrease in salivary secretion rate were also observed in children with obesity, without distinct microbial profiles when compared to normal weight peers^72^. Deciphering causal mechanisms through which the oral microbiota might affect weight gain is outside the scope of this study, especially since we linked microbiota assayed at age two with growth trajectories prior to that age and we have not collected data on oral health/disease. However, the associations we detected should be studied further using data collected longitudinally not just for body size, but also for the microbiota. If confirmed in such a study and for other populations, the relationships we gleaned between the oral microbiota and weight gain could lead to a non-invasive clinical screen to identify children who are at a particular risk of developing obesity later in life. These at-risk children could be closely monitored and be the primary candidates for obesity-prevention interventions^53^.

We also detected a significant association between mothers’ oral microbiota diversity and their children’s growth curves, suggesting the presence of a familial (genetic and/or household) microbiota signature linked to the dynamics of weight gain in early childhood. This too should be explored further, collecting and analyzing longitudinal data on the oral microbiota of parents and children. Also, data on oral cavity diseases should be collected in such future studies, as these conditions may be linked with microbiota and obesity.

In contrast to the oral microbiota, we found that a child’s gut microbiota at age two was not significantly associated with weight gain during the first two years after birth. At first, this appeared surprising, as several studies have linked obesity to decreased diversity and increased F:B ratio in the gut microbiota (see Introduction). However, while an increased F:B ratio is a common marker of obese gut microbiota described in several papers^5,9^ and reviews^3,10^, some studies found no change in Bacteroidetes, increased Bacteroidetes in overweight and lean individuals, or increased Firmicutes in lean patients after gastric bypass (reviewed in^3,10^). Moreover, the obesity signatures of decreased diversity and increased F:B ratio may become pronounced only at later stages of gut microbiota development. This may suggest that the oral microbiota is established with potential signatures of obesity earlier than the gut microbiota. We also note that, despite the lack of significant associations between gut microbiota summary measures and growth curves (or binary weight gain outcome), we did find specific gut taxonomic groups associated with early childhood weight gain. For instance, FLAME identified a Bacteroidetes group and three Firmicutes genera groups in the gut as positively associated with children’s growth curves. These could be pioneers in setting up changes leading to a dysbiosis of gut microbiota associated with increased weight − a hypothesis that should be investigated in future studies.

We collected data on a large number of factors potentially affecting a child’s gut and oral microbiota (see Supplementary Table S2), but with the notable exception of diet-related variables for which we found strong effects on the gut microbiota, their effects were non-significant or they were inconclusive. This could have been due, at least partially, to the small number of subjects in some of the conditions considered. Another potential explanation is that the microbiota were characterized at the age of two, while most factors (except for current diet information) were measured at earlier time points − so their effects might have diluted over time. As already mentioned above, sampling the microbiota at multiple time points during early childhood should alleviate this limitation in future studies and will allow us to further exploit FDA to analyze longitudinal information not only on growth curves, but also on microbiota development.

Interestingly, diet-related variables at the age of two did *not* show significant associations with growth trajectories between birth and two years (no significant association was detected when regressing growth curves on diet categories; Supplementary Table S4). However, perhaps because of the diet variables’ strong effects on the gut microbiota (Table 1), they appear to modulate the relationship between weight gain and the gut microbiota − when adding diet-related variables in a joint regression with the gut microbiota, α-diversity became a significant positive predictor of child growth trajectories (F:B ratio remained non-significant) (Supplementary Tables S5 and S6). In other words, if we compared the gut microbiota of children with the same diet, children who gain weight more rapidly would harbor greater microbial diversity. Thus, a diversity signature in the gut microbiota appears to emerge in our data when controlling for diet, albeit with a sign opposite to the one most commonly identified in the literature − and also opposite to the one we detected in the oral microbiota. We presently do not have an explanation for this finding. FLAME identified a Bacteroidetes group in the gut as positively associated with children’s growth curves, and using linear regression we found that meat consumption had a negative effect on gut F:B ratio (which could be due to a positive association with Bacteroidetes in the denominator). Additionally, meat consumption was significant in the joint functional regression of growth curves on gut F:B ratio and diet-related variables (even though F:B ratio alone was not). The data on this subject are contradictory in the literature. While some researchers found that a high animal protein diet increased gut Firmicutes abundances^73,74^, others found that individuals with a “Western diet” (high in animal protein) have increased gut Bacteroidetes abundances^12,75^. Without question, diet has an important role in shaping the gut microbiota composition and its relationship with body weight. However, more data and more detailed studies are needed on the establishment of the gut microbiota from birth through childhood, and how this relationship affects child growth patterns.

We used two methods to identify influential taxa and it is interesting to note that the taxonomic groups identified by FLAME and LEfSe differed. This could potentially be due to inherent methodological differences between FLAME (a tool that selects groups using high-dimensional function-on-scalar regressions) and LEfSe (a tool using Linear Discriminant Analysis on differentially abundant groups). Alternatively, these incongruences could be due to the very different ways in which the weight gain phenotype were encoded (binary outcome vs. temporal change); and therefore there may be a biological explanation of why bacteria influencing the shape of the growth curves might in fact differ from those discriminating between the microbiota of children with rapid vs. non-rapid infant weight gain. This will require further examination in future studies, as our sequencing data did not allow resolution at the species or strain level, and the functional capacity of the microbiota was unknown.

The use of FDA techniques afforded us the opportunity to associate longitudinal information (growth curves derived from weight and length collected at multiple time points during the first two years after birth) with microbiota measures at the age of two, which, to our knowledge, has not been done before. We used FDA in addition to more traditional statistical analyses based on a binary phenotype (rapid vs. non-rapid infant weight gain). While results on the relationship between growth and microbiota diversity and F:B ratio were consistent between the two approaches, growth curves captured weight gain in a richer fashion than the binary phenotype – leading to stronger results significance. Growth curves also led to additional insights. For instance, a larger number of taxonomic groups were found to be significantly associated with growth curves using FLAME than with rapid vs. non-rapid infant weight gain phenotypes using LEfSe. Our study demonstrated the effectiveness of FDA techniques linking children’s weight gain trajectories and microbiota characterization. This suggests that even greater effectiveness could be achieved in similar studies with longitudinal information also on the microbiota, and indicates the potential of such techniques in a variety of other ‘omics’ applications (e.g.^76^).

## Methods

### Study population

We collected microbiota information on 236 mother-child dyads recruited from the 279 families involved in the INSIGHT study^53^ (Supplementary Fig. S1). These dyads included full-term singletons born to primiparous mothers in Central Pennsylvania, and were predominantly of European descent^64^. The INSIGHT study collected clinical, anthropometric, demographic, and behavioral variables on children and mothers^53^ (Table 1). Parents completed questionnaires reporting children’s dietary intake and exposure to medications. Children’s weight and length were measured at birth, 3–4 weeks, 16 weeks, 28 weeks, 40 weeks, 1 year, and 2 years. For children attending visits before two years, length was measured using a recumbent length board (Shorr Productions). After two years, standing height was measured with a stadiometer (Seca 216).

### Microbiota sample collection

Buccal samples were collected by research staff of the Penn State Hershey Pediatric Clinical Research Office at the child’s two-year clinical research center visit. Information was mailed to participants prior to the visit instructing them to not eat or drink anything, not use tobacco products, and not brush their teeth/use mouthwash for two hours prior to buccal swab collection. Ten sterile cotton swabs were each rubbed for 20 seconds against the inside cheeks of children or mothers. The cotton swabs were placed in tubes containing slagboom buffer^77^. Samples were stored in the Pediatric Clinical Research Office and transported from the Hershey Medical Campus to the University Park Campus where they were processed.

Within two days before the two-year visit, stool samples were collected by parents in stool collection tubes, wrapped in freezer packs, and frozen immediately in the home freezer. They were then brought, packed on ice, to the clinical research site by the parents and stored at −20 °C. Samples were finally transported in coolers on ice from the Hershey Medical Campus to the University Park Campus where they were stored at −80 °C until processing.

### Sample DNA extraction, library preparation, and DNA sequencing

Genomic DNA (gDNA) was extracted from samples using the MoBio PowerSoil DNA isolation kit (Qiagen). The manufacturer’s directions were followed with modifications implemented based on the protocol established by the Human Microbiome Project. These included two heating steps (65 °C and 90 °C for 10 minutes each) prior to bead beating. To control for contamination in the DNA from this stage, a blank ‘sample’ (containing only MoBio Bead Buffer) was subjected to the entire DNA extraction protocol and library preparation. Contamination was never detected via gel electrophoresis, Qubit, or Bioanalyzer.

Library preparation for sequencing of the 16S rRNA gene followed the Illumina protocol ‘16S Metagenomic Sequencing Library Preparation’ (Illumina part# 15044223 Rev.B; https://support.illumina.com/downloads/16s_metagenomic_sequencing_library_preparation.html). Briefly, the variable regions 3 and 4 of the 16S rRNA gene were amplified using PCR (16S_ampliconPCR_For: TCGTCGGCAGCGTCAGATGTGTATAAGAGACAGCCTACGGGNGGCWGCAG and 16S_ampliconPCR_Rev: GTCTCGTGGGCTCGGAGATGTGTATAAGAGACAGGACTACHVGGGTATCTAATCC, PCR conditions are given in the Illumina protocol). The PCR product was isolated using a magnetic bead procedure (Agencourt AMPure XP, Beckman Coulter). Subsequently, Illumina Nextera XT indexes were added and an additional magnetic bead purification was performed. Each library was quantified using a fluorometer (Qubit, ThermoFisher Scientific) and analyzed for correct size on a BioAnalyzer (Agilent). As a positive control, a synthetic mock community DNA pool^78^ was amplified and sequenced alongside the experimental samples. To control for contamination in the amplification or library preparation steps, a blank sample was added to all of the library preparation steps. Contamination was never detected via gel electrophoresis, Qubit, or Bioanalyzer.

An equimolar pool of 48 libraries with a 25% PhiX spike-in was sequenced on an Illumina MiSeq (v3 chemistry, 2 × 300 reads). Demultiplexing was performed using the Illumina software. FASTQ files were retrieved to be used in downstream analyses.

### Sequence analysis

Sequences were first examined using FastQC^79^ and a multi-sample report was generated for textual and graphical views using Galaxy tools^80^ and MultiQC^81^, respectively. FASTQ manipulation filters^80^ were then applied to remove low-quality sequences. Sequences were trimmed using a sliding window approach. To both the 5′ and 3′ ends of the sequences, we applied a cutoff of minimal mean PHRED score of 20 within a window of 5 bases, step size of 1. Quality was reassessed after trimming.

We then merged/joined our forward and reverse paired-end reads into a single contig using a two-step process. First, we used fastq-join from the *ea-utils* package^82–84^ to merge overlapping forward and reverse reads into a single read. This process aligns read pairs and merges overlapping regions based upon user-specified parameters of mismatch percentage and minimum alignment length; we utilized 8% and 6 bases, respectively. To prevent the loss of non-mergeable reads, we performed a second joining operation, using the FASTQ Joiner tool^85,86^ and inserted a string of 5 ambiguous nucleotides (“NNNNN”) between the pairs. We next removed chimeric sequences. We used VSearch^87^ with the uchime_denovo algorithm to create a list of non-chimeric sequences.

Non-chimeric reads were classified individually into taxa. We utilized Kraken^88^ with a customized database containing only 16S rRNA gene sequences, based on GreenGenes^89^. Once Kraken had assigned taxa-kmer counts to individual reads, we utilized a custom abundance reporting tool (https://github.com/blankenberg/Kraken-Taxonomy-Report) to report abundances across samples at specified ranks, along with a phylogenetic tree that was pruned to contain the terminal nodes that are present in at least one of the samples and the connected internal nodes.

### Statistical analyses

#### Diversity measures and tests

Rarefaction and α-diversity calculations were performed using Vegan^56^. Rarefaction was used to normalize the read count across samples - which were all rarefied to 100,000 reads. To compute summary measures of diversity for each microbiota sample, we used the Inverse Simpson α-diversity formula on the phylum level abundance counts (Shannon Diversity Index, Simpson, and Inverse Simpson measures were all highly correlated; see Supplementary Table S9), as computed in Vegan^56^. The Firmicutes-to-Bacteroidetes (F:B) ratio was calculated separately for gut and oral microbiota of each child, and for oral microbiota of each mother, by taking the total count of the number of reads assigned to the Firmicutes phylum divided by the total count of the number of reads assigned to the Bacteroidetes phylum. To test for differences in the proportion of each bacterial phyla between microbiota types (child gut, child oral, mom oral) we used prop.test from the base stats package in R (calculates Pearson’s chi-squared test). For this test we took the average of all individual proportions (calculated as the proportion of reads assigned to that phyla) multiplied by the number of subjects to obtain the “probability of successes”, and to calculate “failures” we took the number of individuals less the “probability of successes”. We compared all possible combinations of the microbiota types (child gut vs. child oral, child gut vs. mom oral, child oral vs. mom oral, and child gut vs. child oral vs. mom oral) and reported Bonferroni-corrected p-values for these 24 tests. Non-metric Multidimensional Scaling analysis, using the Bray-Curtis dissimilarity matrix as the β- diversity measure, was also performed using Vegan^56^. See Supplementary Fig. S6 for a detailed schematic of the computational workflow.

#### Weight outcome and calculation of the the Conditional Weight Gain score

Per the American Academy of Pediatrics^90^, growth measured by weight-for-length is the recommended practice in the United States for children less than two years of age, and BMI becomes the recommended/standard outcome for children who are older than two years. Since we are characterizing child growth from birth to two years, we chose the weight-for-length ratio as our outcome^59^. At each time point when weight and length were collected (see above), we computed the ratio of weight to length (later referred to as growth index). Additionally age- and gender-specific weight- and length-for-age z-scores (WAZ and LAZ, respectively) were determined using the World Health Organization gender-specific child growth standards^91^.

Conditional weight gain (CWG) z-scores were then computed for each child using age- and gender-adjusted anthropometrics at birth and six months^63,64^. Briefly, CWG z-scores were computed as standardized residuals from a linear regression of WAZ at six months on WAZ at birth, using LAZ and precise age at six months visit as covariates. The CWG z-score, therefore, represented the variability of child weight gain explained neither by length at birth and six months nor by gender. By construction, the CWG z-scores had mean 0 and standard deviation of 1. Moreover, in practice these scores were approximately normally distributed. Positive CWG z-scores indicated weight gain that was above the average weight gain, i.e. rapid infant weight gain.

#### Construction of growth curves

The growth indexes calculated above were analyzed longitudinally using tools from Functional Data Analysis (FDA)^92^ alongside the *fda* package in *R*. In particular, individual growth curves were constructed using PACE^60^, a procedure that pools information across subjects to more accurately assemble the curves (Supplementary Fig. S3). The PACE software is freely available for R, and we used it with its default settings. After the curves were assembled, we represented them using 102 cubic spline functions with evenly spaced knots, so that subsequent FDA methods could be applied. Growth curves were also temporally aligned, using the *register.fd* function in *R*, before further analyses were conducted (Fig. 1b).

#### Association between growth curves and microbiota summary measures

We assessed the association between growth curves and α-diversity, as well as the F:B ratio, of gut and oral microbiota fitting *Function-on-Scalar Linear Models*^93^. These were six low-dimensional functional regressions (the growth curve response, i.e. the function, was regressed on a single scalar predictor: α-diversity or F:B ratio), and were carried out in R using code we wrote based on our previous publication^93^. The outcome of these functional regressions were estimated regression coefficient curves, which we obtained using a penalized least squares approach imposing a penalty on the second derivative of the parameter. In each case, the smoothing parameter was set at 10,000, but results were robust against this choice (especially the p-values; see below). The additional smoothing enforced by the penalization was especially useful in terms of producing interpretable estimates. The shape of each estimated coefficient curve indicates how the relationship evolves along the time dimension, with amplitude (distance from zero) and sign (positive or negative) representing strength and direction. Significance of each regression was determined as described in^62^, based on three measures which utilize different types of weighted quadratic forms (note that these were not separate statistical tests, only different ways of determining significance in the same regression). The first, denoted as L2, employs a simple L2 norm (squared integral) of the parameter estimate. The second, denoted as PCA, uses principal components to reduce the dimension of the parameter and then applies a Wald-type test. The last, denoted as Choi, incorporates a weighting scheme into the PCA test so that more principal components can be included, resulting in a test that is in between the PCA and L2 tests, and thus we believe is the preferred measure. We reported results from Choi in the main text, but the results for all three measures of significance can be found in the Supplement. Code is available at: (https://github.com/mreimherr/Insight_Microbiome_Simulation.git).

#### Testing potential co-factors

We tested a wide variety of maternal, health, and behavioral co-factors (gathered by the INSIGHT study)^53^ − a total of 17 (see below) − for effects on the children’s microbiota, and the relationships of the microbiota with children’s weight gain. Maternal gestational weight gain, diabetes during pregnancy, mode of delivery, and gender of the child (4 co-factors) were obtained from electronic medical health records. Maternal smoking during pregnancy, family income, child exposure to antibiotics or acid reducing medications (4 co-factors), were obtained from maternal recall surveys. Intervention group was determined by the INSIGHT study (1 co-factor). First, considering these (non-diet) co-factors one at a time, we tested whether gut and oral microbiota diversity and (separately) F:B ratio differed between their categories using non-parametric Mann-Whitney U and Kruskal-Wallis tests implemented in R (Supplementary Table S2). Specifically, we tested the hypotheses that children with rapid infant weight gain would have (1) a *lower* diversity and (2) a *greater* F:B ratio than children without rapid infant weight gain.

We also obtained information on a child’s diet at two years as reported by parents using an Infant Food Frequency Questionnaire. This questionnaire included 121 food and drink items. Parents reported how often their child had each item in the past week (0, 1, 2–3, or 4–6 times per week, and 1, 2, 3, 4–5, 6 or more times per day). These data were then distilled into a 10-item summary, each item with the corresponding number of weekly consumptions. The items included: sugar-sweetened beverages, milk, dairy (excluding milk), fruit, vegetables, vegetables excluding potatoes, snacks, sweets, meats, and fried foods. We looked at correlations between the consumption frequencies to identify whether any of the items could be removed (we used the R package *Rstats* and the graphical package *corrplot*)^94^. A correlation cut off of 0.7 was employed, eliminating food categories ‘milk’ and ‘vegetables excluding potatoes’ that were highly correlated with other variables. As a result, eight diet categories were retained (8 co-factors). Consumption frequencies for the remaining eight food categories were then used as predictors in four multiple linear regressions for the microbiota summary measures (four regressions in all, for diversity and F:B ratio in children’s oral and gut microbiota; Supplementary Table S3). This was performed using the *lm* function of the *Rstats* package.

Next, we used the *bestglm* package in R^95^ to select the best subset of predictors for a multiple linear regression. This was performed again for four regressions (diversity and F:B ratio in children’s oral and gut microbiota), this time considering 17 covariates at our disposal, as listed in Tables S2 and S3. The covariates selected by *bestglm* were then used as predictors in restricted linear regression fits (results are reported in Table 2 for gut microbiota α-diversity and F:B ratio, for which only diet-related covariates were retained; no covariates were retained by *bestglm* for the oral microbiota summary measures).

Given the prominent effects of diet-related covariates, especially on the gut microbiota (Tables S3 and 2), we also assessed their relationship with weight gain. Specifically, we ran a multiple functional regression for children growth curves (between birth and age two) against food consumption frequencies at age two (Supplementary Table S4; we used the same eight food categories considered in Supplementary Table S3). Finally, we assessed whether diet-related covariates could modulate the relationship between weight gain and the gut microbiota. Specifically, we repeated the two functional regressions of children growth curves on gut α-diversity (Supplementary Table S5) and gut F:B ratio (Supplementary Table S6), in each case adding the diet-related covariates retained by *bestglm* (Table 2). Similar to what we did for the functional regressions for growth curves against microbiota summary measures (see our explanation at the end of the section on ‘Association between growth curves and microbiota summary measures’ of the Methods), statistical significance for these functional regressions was determined with three measures: L2, PCA, and Choi^62^. We reported Choi results in the main text, but retained PCA and L2 results in the supplement.

#### Identification of influential taxonomic groups

To mitigate sparseness and collinearity in our bacterial abundance data, we merged low-abundance or highly correlated genus-level abundance counts into abundances of taxonomic groups. We implemented a two-stage procedure which utilized phylogenetic relationships **–** merging abundances only for neighboring nodes along the phylogenetic tree created from the Kraken taxonomic report tool in Galaxy^88^. First, moving upwards along the tree, we merged a node with its neighbor if its abundance was less than five counts in more than 90% of the samples in the data set. When such a merger occurred, the counts from the two nodes were summed. Next, considering the merged abundances produced by the first stage and moving upwards along the tree, we merged neighboring nodes if their abundances showed a correlation in excess of 0.7 across the samples in the data set. When such a merger occurred the counts were averaged. Notably, this procedure allowed us to tailor the level of resolution of our analyses to the data: branches of the phylogenetic tree where genera were scarcely observed or highly correlated were lumped together, while finer resolution was maintained for branches where genera were more abundant and diversified in their behavior across the samples. The procedure was applied, separately, to abundance data from the three microbiota samples (child gut, child oral and mother oral). Supplementary Table S7 contains a complete list of all taxonomic groups obtained in each microbiota sample.

To identify taxonomic groups with the strongest associations with weight gain, we considered the merged taxonomic group abundances as scalar predictors in functional regressions for growth curves using FLAME (*Functional Linear Adaptive Mixed Estimation*)^66^. Separately, we considered the merged taxonomic group abundances as features in Linear Discriminant Analysis for rapid vs. non-rapid infant weight gain (based on CWG scores) using LEfSe (Linear Discriminant Analysis Effect Size)^67^. These were high-dimensional analyses (each comprised a number of predictors corresponding to the number of taxonomic groups found in a given microbiota sample). The FLAME functional regressions were carried out using methods and R code from^66^. Estimates and p-values were computed using standard methods from FDA (see github repository link below for code) as described in^92^. FLAME simultaneously selected important predictors and captured their effects as estimated regression coefficient curves. It can be thought of as a generalization of the adaptive LASSO^96^ to functional/longitudinal outcomes. In particular, a Sobolev kernel was used in the penalty to produce smooth estimates while also carrying out variable selection. The tuning parameters were chosen via cross validation, as discussed in^66,96^. Code and examples for carrying out the two-stage abundance merger procedure and all of the FDA methods utilized here can be found at https://github.com/mreimherr/Insight_Microbiome_Simulation.git. Standalone code for FLAME is available at: http://personal.psu.edu/~mlr36/codes.html.

### Data Sharing

Raw microbiota reads were deposited in SRA under BioProject number PRJNA420339. Phenotype information was deposited under dbGaP Study number phs001498.v1.p1. All code used is either already public (http://personal.psu.edu/~mlr36/codes.html) or available at GitHub (https://github.com/mreimherr/Insight_Microbiome_Simulation.git). 16S rRNA gene analysis pipeline tools and pipeline are available in the Galaxy platform (usegalaxy.org). The three relevant Galaxy workflows are https://usegalaxy.org/u/sjcarnahancraig/w/16s-qc-3, https://usegalaxy.org/u/sjcarnahancraig/w/kraken-classification, https://usegalaxy.org/u/sjcarnahancraig/w/vegan-rarefacation−alpha-diversity.

### Ethics statement

This study was approved by Penn State University Institutional Review Board (PRAMS034493EP) and all methods were performed in accordance with all relevant guidelines and regulations. Informed consent was received from mothers prior to collection of biological samples and phenotypic, demographic, health, and diet information.

## Electronic supplementary material

Supplementary Information

Supplemental Table 7
