# Supplementary material for: Child Weight Gain Trajectories Linked To Oral Microbiota Composition
Source: Sci Rep. 2018 Sep 19;8:14030. doi: 10.1038/s41598-018-31866-9 (PMC6145887; doi:10.1038/s41598-018-31866-9)
Supplement: Supplementary file 1 — Supplementary Information [file 41598_2018_31866_MOESM1_ESM.pdf]

CHILD WEIGHT GAIN TRAJECTORIES LINKED TO ORAL MICROBIOTA COMPOSITION

Sarah J. C. Craig[1,2], Daniel Blankenberg[3], Alice Carla Luisa Parodi[4], Ian M. Paul[1,5], Leann L. Birch[6], Jennifer S. Savage[7,8], Michele E. Marini[7], Jennifer L. Stokes[5], Anton Nekrutenko[3], Matthew Reimherr*[9], Francesca Chiaromonte*[1,9,10], and Kateryna D. Makova*[1,2]

(1) Center for Medical Genomics, Penn State University, University Park, PA, 16802, USA

(2) Department of Biology, Penn State University, University Park, PA, 16802, USA

(3) Department of Biochemistry and Molecular Biology, Penn State University, University Park, PA, 16802, USA

(4) Department of Mathematics, Politecnico di Milano, Piazza Leonardo da Vinci, 32, Milano, 20133, Italy

(5) Department of Pediatrics, Penn State College of Medicine, 500 University Drive, Hershey, PA, 17033, USA

(6) Department of Foods and Nutrition, 176 Dawson Hall, University of Georgia, Athens, GA, 30602, USA

(7) Center for Childhood Obesity Research, Penn State University, University Park, PA, 16802, USA

(8) Department of Nutritional Sciences, Penn State University, University Park, PA, 16802, USA

(9) Department of Statistics, Penn State University, University Park, PA, 16802, USA

(10) Sant'Anna School of Advanced Studies, Piazza Martiri della Libertà, 33, Pisa, 56127, Italy

***Corresponding authors: Kateryna Makova (kdm16@psu.edu), Francesca Chiaromonte (chiaro@stat.psu.edu), Matthew Reimherr (mreimherr@psu.edu)**

SUPPLEMENTARY INFORMATION

SUPPLEMENTARY TABLES

Table S1. P-values for the simple functional regressions for growth curves against a single microbiota characteristic, considered one characteristic at a time. Each column is a different measure of the significance of the functional regression of the growth curves on the summary measure (see Methods for details[1]**).** Significance of each regression was determined as described in[1], based on three measures which employ different types of weighted quadratic forms (note that these are not separate statistical tests, only different ways of determining significance of the same regression). The first, denoted as L2, employs a simple L2 norm (squared integral) of the parameter estimate. The second, denoted as PCA, uses principal components to reduce the dimension of the parameter and then applies a Wald-type test. The last, denoted as Choi, incorporates a weighting scheme into the PCA test so that more principal components can be included, resulting in a test that is in between the PCA and L2 tests, and thus we believe is the preferred measure. Significant results are in bold.

**Microbiome characteristic/Statistical test**	**L2**	**PC**	**Choi**
Children's oral microbiome α-diversity	0.0414	0.0415	**0.0405**
Children's gut microbiome α-diversity	0.2512	0.2498	0.273
Mothers' oral microbiome α -diversity	0.0148	0.0144	**0.0175**
Children's Oral microbiome F:B ratio	0.0015	0.0015	**0.0015**
Children's gut microbiome F:B ratio	0.8739	0.9235	0.7171
Mothers' oral microbiome F:B ratio	0.0637	0.0626	0.0717

Table S2. Effects of various co-factors (maternal, environmental, and others) on children's microbiota summary measures at the age of two years. Factors considered here are categorical, and do not include diet information (see Table S3). For each, we assessed differences in microbiota summary measures between/across categories using non-parametric tests (one-tailed Mann-Whitney U test for 2 categories, and Kruskal-Wallis test for cofactors for ≥ 3 categories). In all cases, the null hypothesis is that there is no difference between/across categories. Alternative hypotheses and test outcomes (p-values corrected for multiple tests using Bonferroni correction with n=18 tests) are shown in the table, along with sample sizes.

**Factor**	**Microbiota**	**Sample Size**	**α - diversity**	**F:B ratio**
Mom GWG[1]	Child Oral	FailtoMeet = 37<br>Meet = 65<br>Exceed = 112	H_A= medians are different<br>p-value= 1	H_A= medians are different<br>p-value= 1
	Child Gut	FailtoMeet = 29<br>Meet = 59<br>Exceed = 101	H_A= medians are different<br>p-value = 1	H_A= medians are different<br>p-value= 1
Mom smoking during pregnancy	Child Oral	No Smoking = 199<br>Smoking = 15	H_A= Smoking > No smoking<br>p-value = 1	H_A= Smoking > No smoking<br>p-value = 1
	Child Gut	No Smoking = 175<br>Smoking = 14	H_A= Smoking > No smoking<br>p-value = 1	H_A= Smoking > No smoking<br>p-value = 1
Mom diabetes during pregnancy	Child Oral	No Diabetes = 200<br>GD[2] = 11	H_A= GD < No Diabetes<br>p-value = 1	H_A = GD > No Diabetes<br>p-value = 0.55
	Child Gut	No Diabetes = 175<br>GD[2] = 9	H_A = GD < No Diabetes<br>p-value = 1	H_A = GD > No Diabetes<br>p-value = 1
Mode of Delivery	Child Oral	Vaginal = 69<br>C-section = 145	H_A = C-section < Vaginal<br>p-value = 1	H_A = C-section > Vaginal<br>p-value = 1
	Child Gut	Vaginal = 57<br>C-section = 132	H_A = C-section > Vaginal<br>p-value = 0.63	H_A = C-section > Vaginal<br>p-value = 1
Gender	Child Oral	Female = 99<br>Male = 115	H_A= medians are different<br>p-value= 1	H_A= medians are different<br>p-value= 1
	Child Gut	Female = 94<br>Male = 95	H_A= medians are different<br>p-value= 1	H_A= medians are different<br>p-value= 1
Intervention Group	Child Oral	Intervention = 110<br>Control = 104	H_A = Intervention < Control<br>p-value = 1	H_A = Intervention > Control<br>p-value = 1

	Child Gut	Intervention = 98<br>Control = 91	H_A = Intervention < Control<br>p-value = 1	H_A = Intervention > Control<br>p-value = 1
Family Income[3]	Child Oral	1 = 21 ; 2 = 21; 3 = 104; 4 = 60; 5 = 8	H_A = medians are different<br>p-value = 1	H_A = medians are different<br>p-value = 1
	Child Gut	1 = 16; 2 = 17; 3 = 100; 4 = 48; 5 = 8	H_A = medians are different<br>p-value = 1	H_A = medians are different<br>p-value = 1
Antibiotic (Abx) exposure birth to 2 years	Child Oral	No Abx = 80<br>Abx = 133	H_A = Abx < No Abx<br>p-value = 1	H_A = Abx > No Abx<br>p-value = 1
	Child Gut	No Abx = 70<br>Abx = 118	H_A = Abx < No Abx<br>p-value = 1	H_A = Abx > No Abx<br>p-value = 1
Acid reducing medicine exposure birth to 2 years	Child Oral	No ARD[4] = 197<br>ARD = 16	H_A = ARD < No ARD<br>p-value = 1	H_A = ARD > No ARD<br>p-value = 1
	Child Gut	No ARD = 177<br>ARD = 12	H_A = ARD < No ARD<br>p-value = 1	H_A = ARD > No ARD<br>p-value = 1

[1]GWG - Gestational Weight Gain

[2]GD = Gestational Diabetes (controlled with exercise and diet)

[3]Family Income categories: 1= <$24,999; 2= $25,000 - 49,999; 3= $50,000 - 74,999; 4= ≥$100,999; 5= don’t know or refuse to answer

[4]ARD = Acid Reducing Drug

Table S3: Results for four multiple linear regressions linking microbiota and diet. Each of our two summary measures, α-diversity and Firmicutes-to-Bacteroidetes ratio, separately in children's oral (n=196) and gut (n=175) microbiota at age two, were regressed against diet-related covariates expressing consumption of various categories of food at the same age. Coefficient estimates and significance (p-values) are shown for each covariate in each regression. P-values for overall model significance are shown in the last row. Significant results are in bold.

	**Oral α -diversity**		**Oral F:B**		**Gut α -diversity**		**Gut F:B**	
	**Coefficient**	**p-value**	**Coefficient**	**p-value**	**Coefficient**	**p-value**	**Coefficient**	**p-value**
**SSB**[1]	-0.03342	0.582	1.1275	0.3841	0.08336	0.05528	6.2883	0.175894
**Dairy**	-0.03352	0.173	-0.54648	0.2967	0.01577	0.37724	1.3896	0.467484
**Fruit**	0.02779	0.252	-0.05704	0.9119	**0.05566**	**0.00132**	0.3566	0.845388
**Veg**[2]	-0.01400	0.666	**1.68720**	**0.0153**	**-0.06882**	**0.00434**	**15.1390**	**1.55e-08**
**Snacks**	0.02184	0.751	-0.56059	0.7024	0.01716	0.71927	5.6731	0.267925
**Sweets**	0.07251	0.426	-1.32147	0.4953	-0.10886	0.08751	-5.8599	0.388992
**Meats**	0.07157	0.361	-2.40060	0.1505	0.01061	0.84823	**-21.2666**	**0.000432**
**Fried**	-0.02442	0.880	4.39098	0.2052	0.16431	0.15996	12.3237	0.324406
**Overall Model**		0.7042		0.2612		**2.63×10^{-3}**		**1.65×10^{-7}**

1: SSB = Sugar Sweetened Beverages

2: Veg = Vegetables

Table S4. P-values for the multiple functional regression of growth curves with eight (considered simultaneously) diet-related covariates at age two. Each row shows a different food category, and each column a different measure of significance for the same functional regression of the growth curves on the levels of consumption observed for the food categories (see Methods for details[1])**.** See the legend to Supplementary Table 1 for the explanation of the three significance measures.

**Diet category/ Statistical test**	**PC**	**Choi**	**L2**
**2 year_SSB[1]**	0.997	0.483	0.767
**2 year_Dairy**	0.743	0.761	0.763
**2 year_Fruit**	0.739	0.684	0.739
**2 year_Veg[2]**	0.564	0.509	0.556
**2 year_Snacks**	0.711	0.838	0.745
**2 year_Sweets**	0.156	0.173	0.158
**2 year_Meats**	0.0968	0.0899	0.0952
**2 year_Fried**	0.275	0.22	0.263

1: SSB = Sugar Sweetened Beverages
2: Veg = Vegetables

Table S5. P-values for the multiple functional regression of growth curves with gut α-diversity and two diet-related covariates (fruit and vegetable consumption) at age two (these three variables are considered simultaneously in the regression). The rows show the three predictors used, and each column shows a result of a different assessment of a p-value for the same functional regression of the growth curves on such predictors (see Methods for details;[1])**.** See the legend to Supplementary Table 1 for the explanation of significance measures. Significant results are in bold.

		L2	PC	Choi
Child Gut	Fruit	0.2834	0.2883	0.2809
	Veg	0.6379	0.7540	0.4117
	diversity	0.1574	0.2413	**0.0200**

Table S6. P-values for the multiple functional regression of growth curves with gut Firmicutes-to-Bacteroidetes and diet-related covariates at age two. See legend to Supplementary Table S5.

		L2	PC	Choi
Child Gut	Veg	0.47382	0.79306	0.13682
	Meat	**0.00641**	**0.00694**	**0.00589**
	F:B	0.63214	0.71746	0.48127

Table S7. Taxonomic groups and their composition. This table describes the result of the two-stage merging procedure (see Methods “Identification of influential taxonomic groups”) to aggregate sparse and highly-collinear abundances for each microbiota type (child gut, child oral, and mom oral).

SEE EXCEL FILE

Table S8. Taxonomic groups (and the genera they contain) identified by FLAME and LEfSe as informative for either growth curves (FLAME) or rapid vs. non-rapid weight gain (LEfSe).

**Groups identified with FLAME**		
**Child Gut Groups**	12	*Porphyromonas, Tannerella*
	23	*Turicibacter*
	26	*Dehalobacterium, Christenesenella, Caldicoprobacter, Heliorestis, Lutispora, Gracilibacter*
	41	*Anaerotruncus, Anaerofilum, Ethanoligenens Dialister, G07*
	61	*Zymomonas, Sphingopyxis, Sphingomonas, Sphingobium, Novosphingobium, Kaistobacter, Blastomonas, Lutibacterium, Erythrobacter, Wolbachia, Rickettsia, Neorickettsia, Ehrlichia, Candidatus Neoehrlichia, Anaplasma, Telmatospirillum, Skermanella, Roseospira, Rhodovibrio, Rhodospirillum, Phaeospirillum, Novispirillum, Nisaea, Magnetospirillum, Inquilinus, Azospirillum, Swaminathania, Roseomonas, Roseococcus, Gluconobacter, Acidocella, Acidisphaera, Acidiphilium, Acetobacter, Thalassobius, Shimia, Sagittula, Ruegeria, Rubellimicrobium, Rhodovulum, Rhodobacter, Rhodobaca, Phaeobacter, Paracoccus, Octadecabacter, Nautella, Marivita, Loktanella, Dinoroseobacter, Antarctobacter, Anaerospora, Amaricoccus, Oceanicaulis, Maricaulis, Hyphomonas, Hirschia, Xanthobacter, Labrys, Blastochloris, Azorhizobium, Afifella, Sinorhizobium, Shinella, Rhizobium, Kaistia, Candidatus Liberibacter, Agrobacterium, Phyllobacterium, Nitratireductor, Mesorhizobium, Defluvibacter, Chelativorans, Aminobacter, Pleomorphomonas, Methylosinus, Methylopila, Methylobacterium, Rhodoplanes, Rhodobium, Pedomicrobium, Parvibaculum, Hyphomicrobium, Devosia, Cohaesibacter, Pseudochrobactrum, Ochrobactrum, Nitrobacter, Bradyrhizobium, Bosea, Balneimonas, Afipia, Methylocella, Chelatococcus, Beijerinckia, Bartonella, Martelella, Fulvimarina, Aurantimonas, Thalassospira, Phenylobacterium, Mycoplana, Caulobacter, Brevundimonas, Asticcacaulis, Caminibacter, Sulfurimonas, Sulfuricurvum, Helicobacter, Flexispira, Sulfurospirillum, Campylobacter, Arcobacter, Mariprofundus*
**Child Oral Groups**	5	*Rhodococcus, Nocardia, Mycobacterium, Virgisporangium, Verrucosispora, Solwaraspora, Pilimelia, Dactylosporangium, Couchioplanes, Catellatospora, Actinoplanes, Actinocatenispora, Propionicimonas, Pimelobacter, Nocardioides, Kribbella, Friedmanniella, Aeromicrobium, Actinopolymorpha, Streptomonospora, Prauseria, Nocardiopsis, Xylanimicrobium, Promicromonospora, Cellulosimicrobium*
	13	*Parabacteroides*

**Mother Oral Groups**	28	*Trichococcus*
	53	*Fusobacterium, Cetobacterium, Propionigenium, Psychrilyobacter, u114*
**Groups identified with LEfSe**		
**Child Gut Groups**	4	*Bifidobacterium, Alloscardovia, Bombiscardovia, Gardnerella, Scardovia*
	46	*Dialister, G07*
**Child Oral Groups**	14	*Porphyromonas*
	21	*Anoxybacillus, Anaerobacillus, Alkalibacillus*
	25	*Jeotgalicoccus, Macrococcus, Salinicoccus*
	33	*Granulicatella*
	35	*Enterococcus, Tetragenococcus, Vagococcus*
	45	*Anaerovibrio, Anaeromusa, Acidaminoccus, BSV43*

Table S9: Correlations between Shannon Diversity Index, Simpson diversity measure, and Inverse Simpson Diversity measure[2].

**Child gut**	Inverse Simpson	Shannon	Simpson
Inverse Simpson			
Shannon	.95		
Simpson	.95	.95	
**Child oral**			
Inverse Simpson			
Shannon	.95		
Simpson	.94	.98	
**Mother oral**			
Inverse Simpson			
Shannon	.93		
Simpson	.93	.98	

SUPPLEMENTARY FIGURES

Figure S1. Overall summary of data collection and samples. (a) Description of participant enrollment into the study and collection of samples. (b) Summary of the 215 maternal samples, 214 child oral samples, and 189 child gut samples showing how many samples of each type were collected from the same family (e.g., for 169 families we collected samples of all three types).

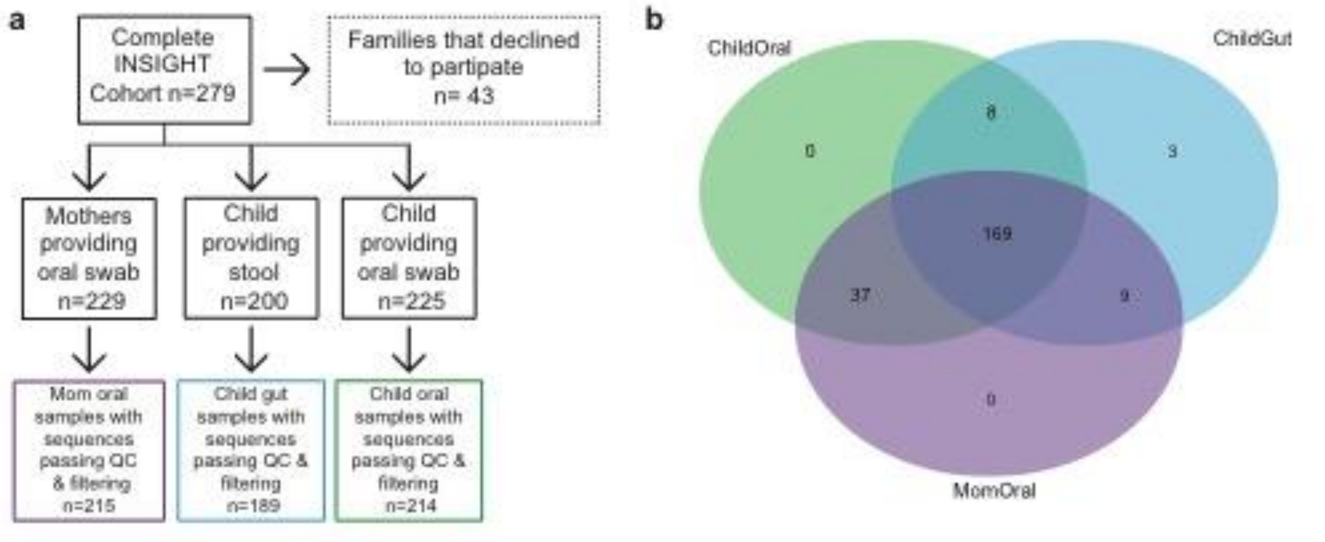

Figure S2. The distribution of phyla in the oral and gut communities of two-year old children and their mothers. (a) Bar plot depicting the proportions of different bacterial phyla in each microbiota type.The proportions of phyla collapsed into “OTHER” were below 1% of the abundances in more than 90% of the samples. Error bars represent the standard deviation from the mean. P-values are corrected for multiple testing using the Bonferroni correction with 24 tests. (b) A non-metric multidimensional scaling (NMDS) plot using Bray-Curtis dissimilarity values calculated from Phyla level classification[3] (see Supplemental Methods for details) between child oral, child gut, and maternal oral microbiomes. Ellipses are drawn to show the 95% confidence around the centroid (the mean position of all points from a sample).

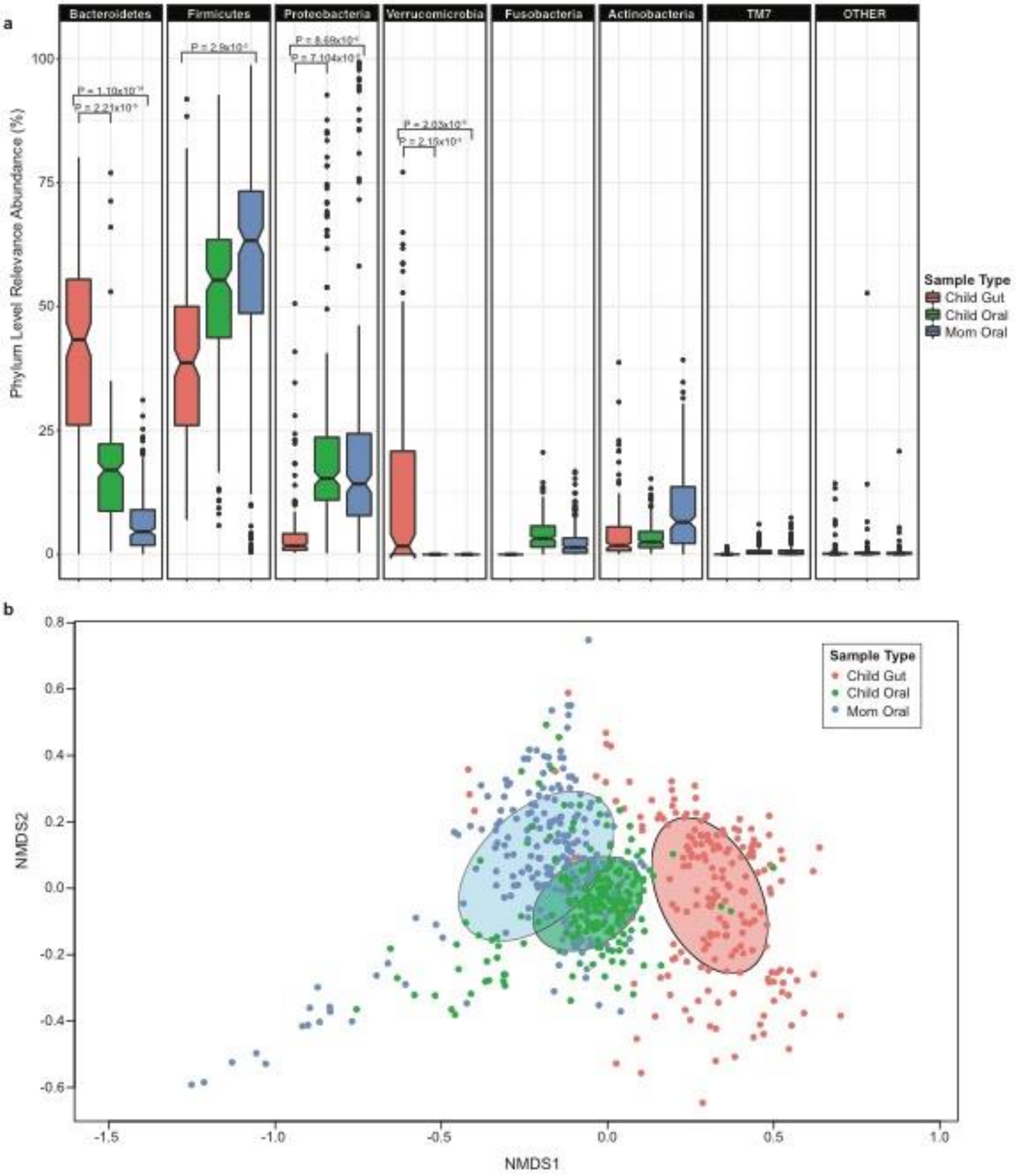

Figure S3. Construction of growth curves using functional data analysis (a) Example growth curves for ten individuals. Points: observed weight-to-length ratios (i.e. growth indices); lines: estimated growth curves. (b) Growth curves for 226 children prior to alignment (the same curves after alignment are shown in Fig. 1B).

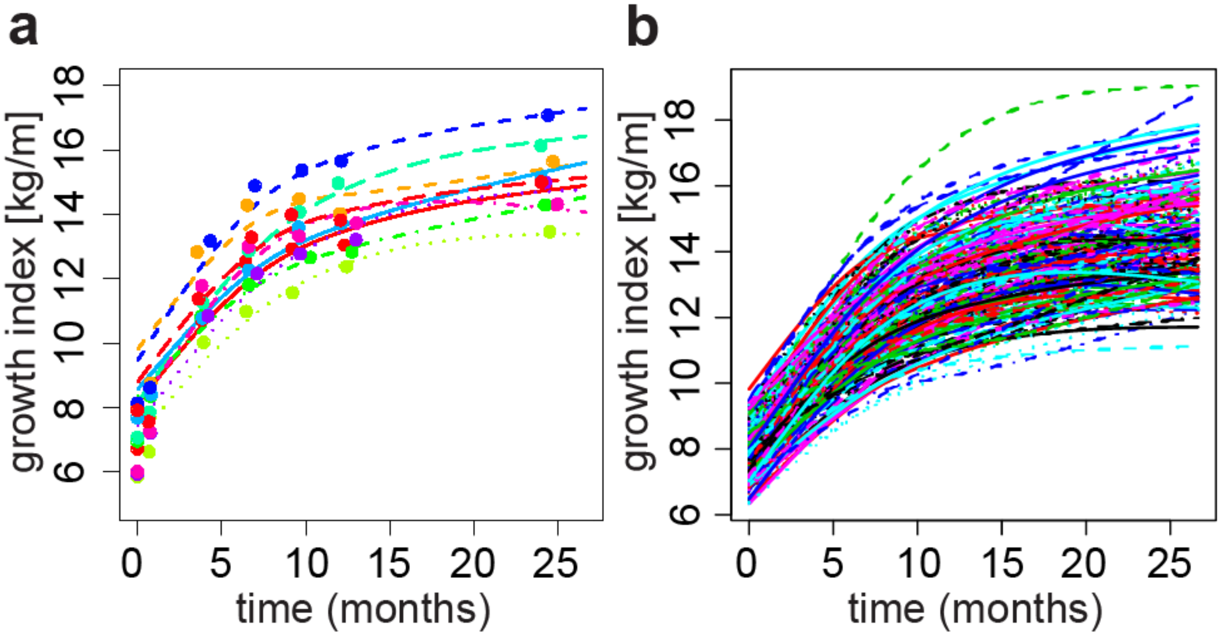

Figure S4. Box plot comparing the weight at 2 years in children with rapid weight gain (CWG ≥ 0) vs. children without rapid weight gain (CWG < 0). CWG ≥ 0 n = 103; CWG < 0 n = 123; P-value = 4.627e-13, one-tailed Mann Whitney U test.

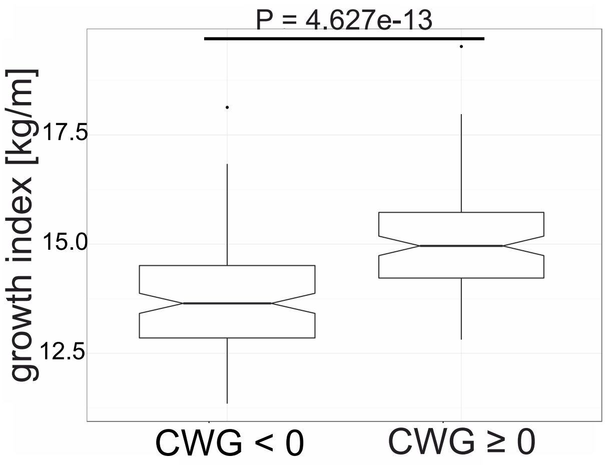

Figure S5. Gut microbiota's relationships with growth curves, accounting for diet at age two. Estimated regression coefficient curves expressing the associations of growth curves with the gut microbiome diversity (a) and F:B ratio (b) when diet-related covariates are added to the two multiple functional regressions. Each curve is accompanied by a point-wise confidence band.

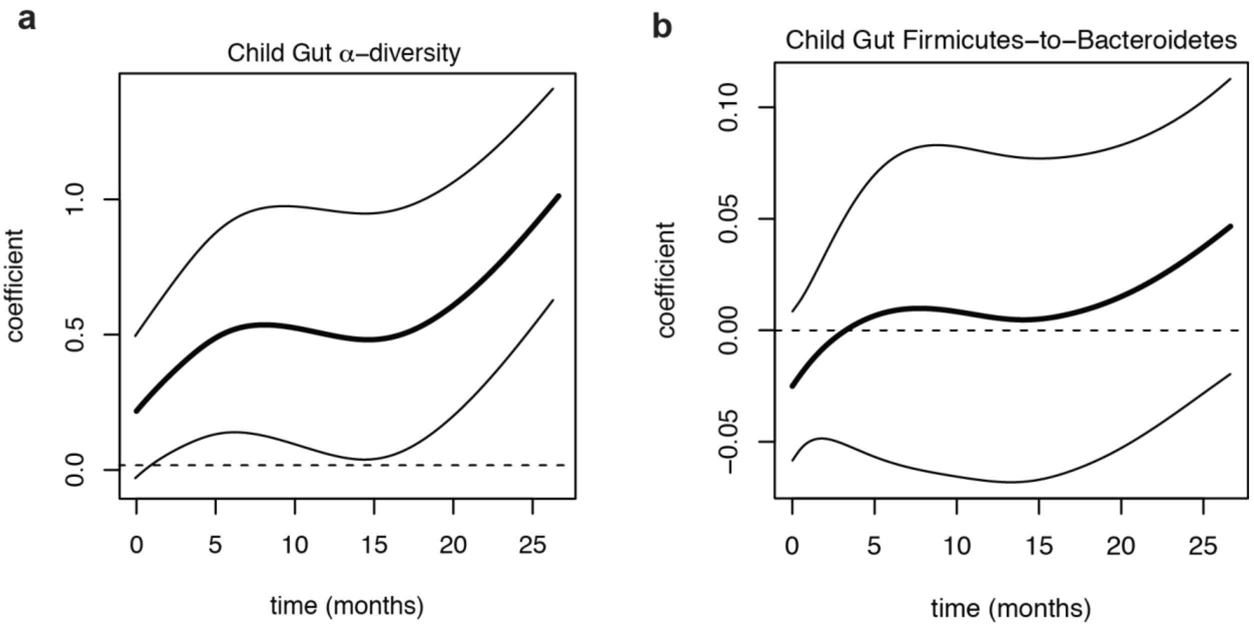

Figure S6. Schematic of Galaxy 16S rRNA gene workflow. The workflow can be subdivided into three broad stages: 1) Quality control (shown in blue outlines), 2) Classification (shown in green outlines), and 3) Downstream analysis (orange outlines)

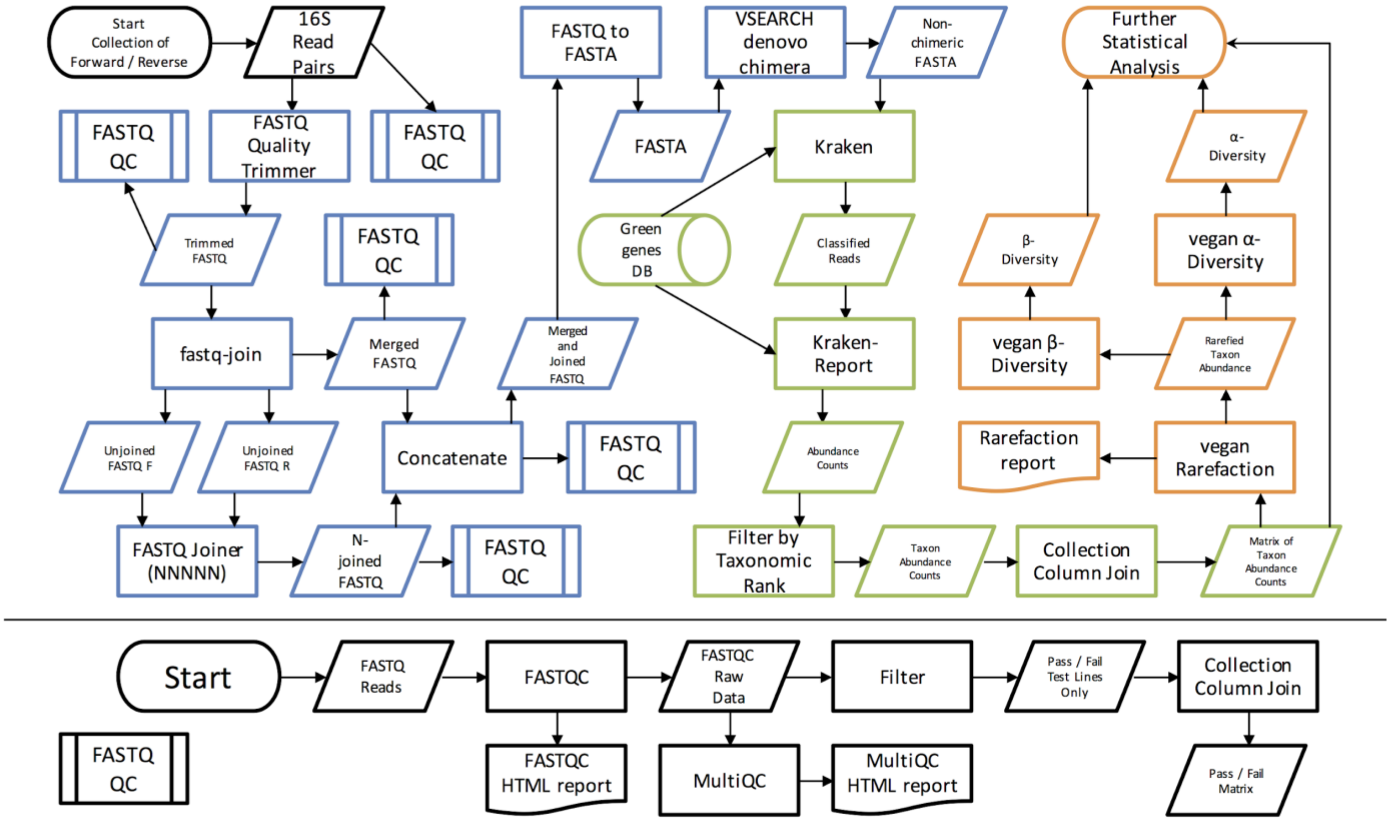

REFERENCES

1. Choi, H. & Reimherr, M. A Geometric Approach to Confidence Regions and Bands for Functional Parameters. (2017).
2. Whittaker, R. H. Evolution and Measurement of Species Diversity. *Taxon* **21,** 213 (1972).
3. Koleff, P., Gaston, K. J. & Lennon, J. J. Measuring beta diversity for presence–absence data. *J. Anim. Ecol.* (2003).
